# Single-Molecule Conductance through Hybrid Radially and Linearly π‑Conjugated Macromolecules Reveals an Unusual Intramolecular π‑Interaction

**DOI:** 10.1021/acs.nanolett.5c03693

**Published:** 2025-07-24

**Authors:** Wanzhuo Shi, Mengjiao Wang, Latha Venkataraman, John D. Tovar

**Affiliations:** † Department of Chemistry, 5798 Columbia University, New York, New York 10027, United States; ‡ 148492 Institute of Science and Technology Austria, 3400 Klosterneuberg, Austria; § Department of Chemistry, 1466 Johns Hopkins University, Baltimore, Maryland 21218, United States; ∥ Department of Applied Physics and Applied Mathematics, 5798 Columbia University, New York, New York 10027, United States; ⊥ Department of Materials Science and Engineering, 1466 Johns Hopkins University, Baltimore, Maryland 21218, United States

**Keywords:** Single-Molecule Junctions, Radial Conjugation, van der Waals Interaction, Molecular Conformation

## Abstract

We describe the design, synthesis, and single-molecule junction conductance of π-electron molecules bearing both radial and linear π-conjugation pathways, whereby cycloparaphenylene (CPP) radial cores are π-extended linearly with aryl alkyne substituents as models for previously reported CPP-arylene ethynylene conjugated polymers. Although radially and linearly conjugated molecules have been studied previously in isolation as junction-bridging molecular electronic units, this is the first study to examine molecules where both topologies are operative. Our results reveal that the presence of radial CPP components within the junction-spanning pathway leads to a reduction in the conductance of the backbone compared to model linear phenyl substituents. Through tight-binding and DFT-based calculations, we attribute this conductance change to intramolecular van der Waals (vdW) interactions between the CPP ring and the junction-spanning arylene-ethynylene molecular backbone. These interactions induce changes in the dihedral angles of the backbone, leading to a reduced overlap of π orbitals within the molecular junction.

## Graphical abstract

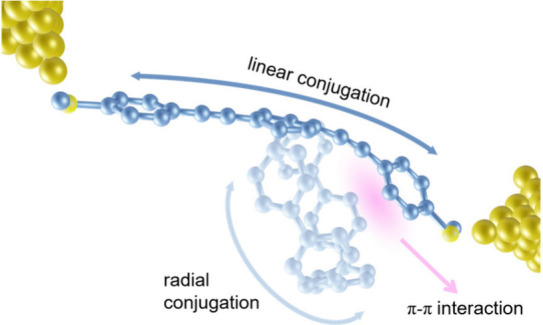

Cycloparaphenylenes (CPPs) are a class of intriguing macrocycles possessing radial conjugation, with well-defined molecular structures that represent small fragments of carbon nanotubes.^1^ Radial conjugation has elicited interest from chemists for decades, with some of the first reported examples in the 1990s being Diederich’s cyclocarbons,^2^ Oda’s phenylene ethynylene macrocycles,^3^ and Herges’ picotubes.^4,5^ CPPs have since risen to the forefront on account of their scalability (gram-scale synthesis)^6–8^ and variability of ring size and composition, thus enabling vast molecular property tuning and offering many new physiochemical explorations and extensions to materials science.^9–12^ We became interested in understanding how the unique radial conjugation offered by the CPP unit would impact the π-electron delocalization within traditionally linearly conjugated organic semiconducting oligomers and polymers. Our initial results revealed that the merger of radial and linear conjugation offers new electronic structures and transport opportunities that are not simply additive superpositions of the contributions from the two different conjugation pathways.^13,14^

To further this exploration, we sought to probe the conductance of defined small molecules bearing both radial and linear conjugation pathways at the single molecule level using the scanning-tunneling microscope-based break-junction setup (STM-BJ), a recognized state-of-the-art method for single molecule conductance interrogation.^15–19^ CPPs on their own have been subject to STM-BJ interrogation previously, revealing multiple important insights. First, owing to the radially oriented π-cloud that surrounds a CPP, an Au STM tip can form a contact with the ring through a comparatively weak π-interaction, so the typical Au linkers that can form covalent/dative bonds are not strictly required (such as thiols/thiomethyls). Second, the conductance progressively decreases as the CPP ring size increases, although in general the CPPs maintain unusually large tunneling coefficients (β-values) and large conductances for structures of their size. Third, the application of larger junction biases provokes controlled degradation chemistry whereby the CPP undergoes a putative electrophilic aromatic substitution reaction with the gold STM tip followed by facile bond scission and CPP ring opening into a linear phenylene oligomer spanning the junction.^20,21^ These prior findings, while important, do not inform on the global impact of radial and linear π-conjugation on the observed transport in a permanently intact system. In this study, we examine the single-molecule junction conductance through molecules bearing both radially conjugated CPPs and linearly conjugated aryl acetylenes with terminal thiomethyl groups using the STM-BJ setup in order to learn how the merger of both topologies affects transport properties. Our experimental results demonstrated that compared to benzene substituents, the presence of CPP rings reduces the conductance of the main chain. Using tight-binding models, we attributed this conductance reduction to a decrease in the π-orbital overlap along the main chain. Further density functional theory-based (DFT) calculations revealed that the change in π-orbital overlap arises from the intramolecular vdW interactions between the CPP ring and the aryl acetylene backbone.

In our prior work, we utilized arylene ethynylene monomers as partners for Pd-catalyzed Sonogashira polymerizations to prepare conjugated polymers, where the arylene unit was a radially conjugated CPP unit or a linearly conjugated terphenyl as a nonradial control.^14^ We saw these as precursors for thiol-terminated arylene ethynylene oligomers all bearing the same π-conjugated backbone: 1,4-bis­((4-(methylthio)­phenyl)­ethynyl)­benzene, which has been the subject of several prior studies.^22,23^ Thiomethyl anchor groups allow these molecules to bind to Au electrodes, forming single-molecule junctions. In these derivatives, the central phenyl ring is extended to oligomeric aromatic structures of varying sizes: linear terphenyl (**T3**) along with radial [6]­CPP (**C6**) and [8]­CPP (**C8**). The terphenyl model allows us to capture the local electronics imposed by the CPP but without the curvature while the [6] and [8]­CPPs allow us to probe any ring-size/diameter effects. The molecular structures are shown in Scheme 1, and the synthesis procedures are detailed in the SI Section 1. The [6]­CPP and [8]­CPP dialkynes **1** and **2** were prepared previously according to the well-established building block approach developed by Jasti to gradually build the strain of macrocycles utilizing cyclohexadiene moieties as curved pro-aromatic rings and install the alkyne handles in a final macrocyclization.^13,24^ Thiomethyl anchor groups in **C6** and **C8** were then introduced through Sonogashira coupling reactions between (4-iodophenyl)­(methyl)­sulfane and the [6] and [8]­CPP dialkynes, respectively (Scheme 1). The linear terphenyl model molecule **T3** was also obtained through Sonogashira coupling between the dialkynylated terphenyl **3** and iodinated thioanisole.

**Scheme 1:**
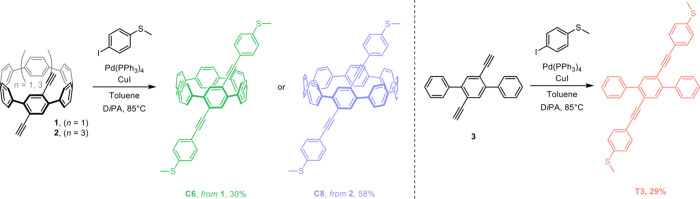
Synthesis of **C6**, **C8**, and **T3** via Sonogashira Couplings

We measure the single-molecule conductance using the scanning tunneling microscope-based break-junction (STM-BJ) technique.^15,16^ For each molecule, we prepare 100 μM solutions in 1,2,4-trichlorobenzene (TCB) and propylene carbonate (PC, data shown in Figure S1) and perform measurements at a tip bias of 100 mV. We repeatedly advance and retract the tip to the substrate while continuously recording conductance-displacement traces throughout the process. Figure 1 presents one-dimensional (a) and two-dimensional (b, c, d) histograms, logarithmically binned and constructed without data selection from over 3000 individual conductance-displacement traces measured in TCB. The sharp peak at 1 *G*_0_ (*G*_0_ = 2*e* ^2^/*h*, the conductance quantum) confirms the formation of Au point contacts. The additional plateaus observed below the metallic conductance correspond to transport through the Au–molecule–Au junctions.

**Figure 1:**
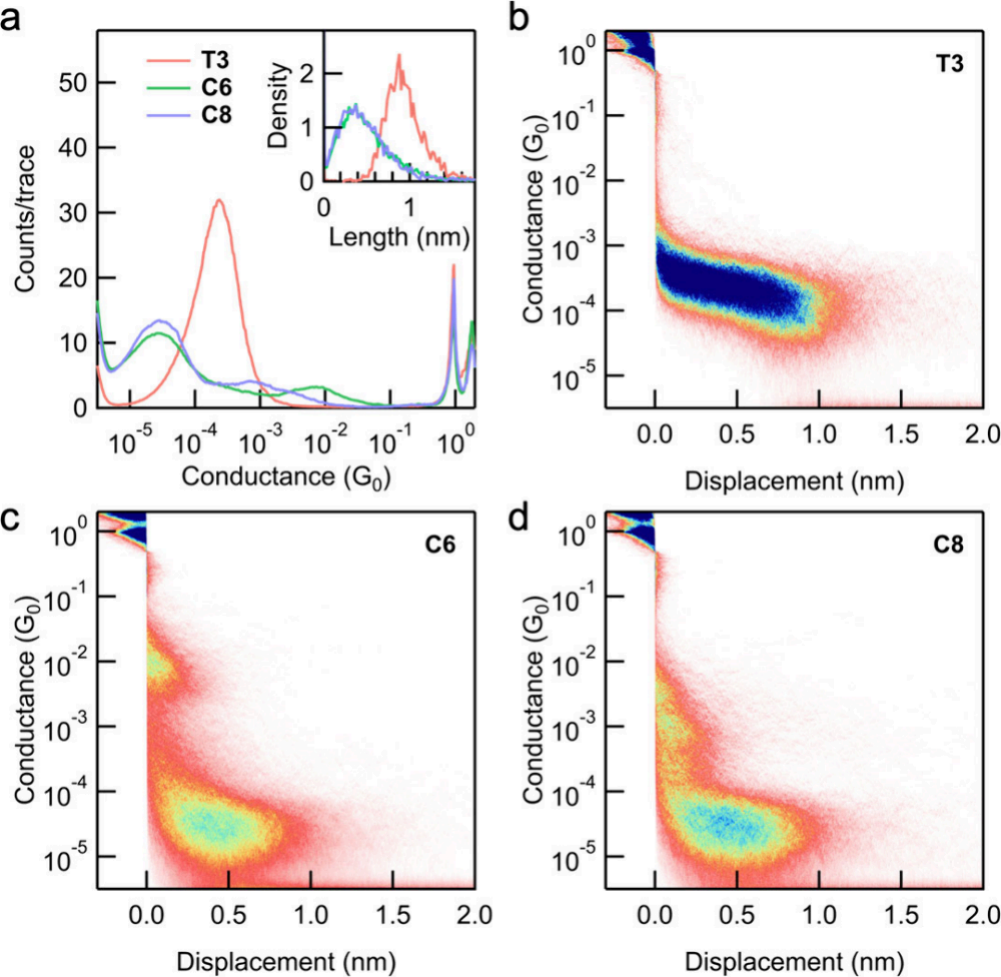
(a) Logarithmically binned 1D conductance histograms for **T3**, **C6**, and **C8**. Each histogram is generated from over 3000 traces without selection. Inset: normalized molecular plateau length distribution histograms. The corresponding 2D conductance-displacement histograms for **T3** (b), **C6** (c), and **C8** (d) are shown.

To determine the most probable conductance values for the target molecules, we fit the molecular peaks with a Gaussian function. The conductance peak of **T3** is at 2.3 × 10^–4^ *G*_0_, whereas **C6** and **C8** both have conductance peaks at 2.7 × 10^–5^ *G*_0_, which is almost 1 order of magnitude lower. This significant difference in conductance is unexpected, as these derivatives share the same phenylene-ethynylene charge transport pathway and all the side groups are phenylene-based structures which lack strong electron-withdrawing or -donating properties and are attached at identical positions with respect to the bridging junction.

To investigate how different side groups influence junction formation, we analyzed the step-length distributions for all three molecules. The step-length of each junction is defined as the distance from the breaking of the Au–Au point contact to the rupture of the Au–molecule–Au junction, identified by the conductance dropping to the noise floor. The probability density histograms, shown in the insets of Figure 1a, reveal that the junction length of **T3** is significantly longer than those of **C6** and **C8**, which display nearly identical distributions. We hypothesize that the bulky CPP ring imposes steric constraints, reducing the probability of junction formation and resulting in shorter junction elongation lengths. These length differences are also clearly visible in the two-dimensional histograms shown in Figure 1b–d where **T3** shows a long plateau while both **C6** and **C8** show a shorter feature with a peak distribution around 0.5 nm.

Given that CPP rings can also bind to Au electrodes through vdW interactions, we conducted conductance measurements on the **Half-C8** molecule, which contains only one thiomethyl group. The molecular structure and the conductance histograms are presented in the Figure S2. The absence of the conductance peak near 10^–5^ *G*_0_ in these measurements confirms that the electron transport pathway requires the presence of two thiomethyl terminal groups.

We note a second, less-intense peak around 1 × 10^–2^ *G*_0_ for **C6** and 1 × 10^–3^ *G*_0_ for **C8**. By contrast, the dominant lower-conductance peak discussed above arises from junctions in which both thiomethyl groups bind to the electrodes. To test whether the higher-conductance feature bypasses the sulfur contacts, we measured a control [6]­CPP molecule that contains no thiomethyl substituents. Its 1D and 2D histograms (see Figure S3) show a broad peak centered near 1 × 10^–2^ *G*_0_, matching earlier reports of Au–CPP–Au “π-complex” junctions.^20,21^ In addition, we examined the noise associated with the high-conductance plateau of **C6** and benchmarked it against that of [6]­CPP. The resulting noise-conductance scaling confirms that charge transport in that junction proceeds predominantly through-space tunneling (detailed in SI Section 4). Thus, we attribute the additional high-conductance peaks for **C6** and **C8** to Au–CPP–Au junctions stabilized by vdW and π-complex interactions.

To understand the charge transport mechanism and rationalize the conductance data, we employ a tight-binding model to simulate these conjugated systems. Although this model is a significant simplification compared to one based on density functional theory, it often captures the essential features.^25,26^ More importantly, it allows us to adjust parameters on demand, enabling us to identify the origins of conductance variations across this series. To simulate the electron conduction in single-molecule junctions, the transmission function is commonly calculated by Green’s function method, written as^27,28^

1 $$\begin{array}{c} T\left(E\right)=Tr\left[\Gamma_{L}G\Gamma_{R}G^{†}\right] \end{array}$$

where *G* and *G* ^†^ represent the retarded and advanced Green’s functions, respectively, while Γ_*L*_ and Γ_*R*_ are matrices that characterize the coupling to the left and right electrodes, with the wide-band limit assumed.^29,30^ The Green’s function of matrix form in atom basis can be written as^27,28^

2 $$\begin{array}{c} G\left(E\right)={\left[EI-H_{\mathrm{mol}}+\frac{i\left(\Gamma_{L}+\Gamma_{R}\right)}{2}\right]}^{-1} \end{array}$$

where *I* is the identity matrix and *H*_mol_ is the Hamilton of the isolated molecule using an atomic basis. Using the Hückel model, all carbon atoms are included, and their on-site energies are set to zero for simplicity. The coupling strength of C–C bonds is described using the hopping integral *t*, where stronger interactions are represented by more negative values. For phenyl rings, the hopping integral is set to −1, while single bonds in the molecular backbone are assigned −0.8, and triple bonds are set to −1.2. The full description of the tight-binding model is in the SI Section 5.

We calculated the transmission functions for all three molecules using the tight-binding model; these are shown in Figure 2a. The transmission of **T3** is symmetric due to the zero on-site energy used for all atoms. Introducing a CPP ring as the side structure generates multiple additional resonances, corresponding to the orbitals of the isolated CPP ring. These appear as pairs of sharp peaks and dips. Notably, two new orbitals are introduced between the HOMO and LUMO of **T3**, and their energy positions are pinned, regardless of the size of the CPP ring. Interestingly, despite the significant impact on the shape of the transmission function, the transmission values at the Fermi level—corresponding to the low-bias conductance—are the same for all three molecules.

**Figure 2:**
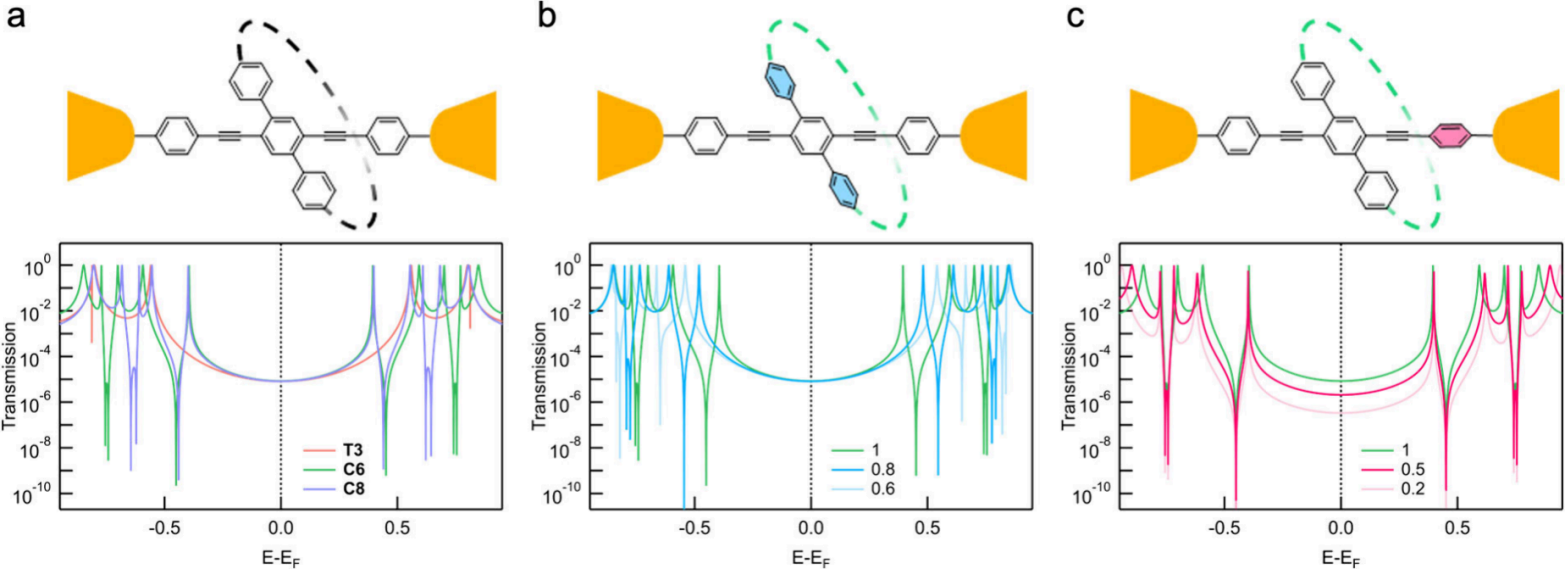
(a) Tight-binding modeled transmission functions of **T3**, **C6**, and **C8**. The scheme of the modeled structures is shown in the top panel. (b) Effect of the scaling factor (*s*_CPP_) on the orbital overlap between the phenyls and the CPP cycle. *s*_CPP_ = 1 represents the original **C6** model. (c) Effect of the scaling factor (*s*_Backbone_) on the orbital overlap between the phenyls on the conduction backbone. *s*_Backbone_ = 1 represents the original **C6** model.

To explore the origin of the unexpected conductance differences observed in experiments, we adjusted the hopping integrals for various bonds to identify the key factors that alter transmission at the Fermi level in **C6**. First, we focused on the dihedral angles between the phenylene rings within the macrocyclic CPP unit. As previously discussed, the CPP ring can constrain the dihedral angle between phenylenes, which we quantified by applying a scaling factor (*s*_CPP_ < 1) to the hopping integral. Interestingly, while this caused shifts in the energetic positions of the resonances, the transmission value at the Fermi level remained unchanged, as shown in Figure 2b. This indicates that the flexibility of the CPP macrocycle affects the energy of the orbitals localized on them but does not impact the transmission along the junction-bridging molecular backbone near the Fermi level.

Next, we examine the effect of reducing orbital overlap between the phenylene groups along the conduction backbone. It is well established that twisting aromatic rings out of planarity diminishes their π-orbital overlap and conjugation, leading to lower conductance.^16,31,32^ This can be modeled by introducing a scaling factor (*s*_Backbone_ < 1) to the hopping integrals along the backbone. As shown in Figure 2c, adjusting this factor leaves the positions of the resonances induced by the CPP ring unchanged. However, the transmission value at the Fermi level decreases. We hypothesize that in the experiments, the CPP ring affects orbital overlap along the conduction pathway through modifying the planarity of the backbone, resulting in a conductance difference of approximately 1 order of magnitude observed in experiments.

To better understand the features captured by the tight-binding model and identify the origin of the reduced π orbital overlap along the molecular backbone, we carried out DFT-based transmission calculations for the molecular series.^33–38^ We used the B3LYP functional for all DFT related calculations in this work.^39–41^ The calculation details are provided in the SI Section 5.

We first performed the calculation without including any vdW corrections. The junction geometry for a **C6** molecule attached to two 22-atom Au electrodes through Au–S donor–acceptor bonds is shown in Figure 3a. The structures of **T3** and **C8** are provided in Figure S4. The three phenylene rings in the conduction pathway are nearly coplanar, suggesting strong π orbital overlap along the backbone. The resulting transmission functions of all three molecules are shown in Figure 3c. The transmission values at the Fermi level—which correspond to the conductance measured at low bias—are nearly identical. This finding is consistent with the results from tight-binding toy model but does not align with the experimental data.

**Figure 3:**
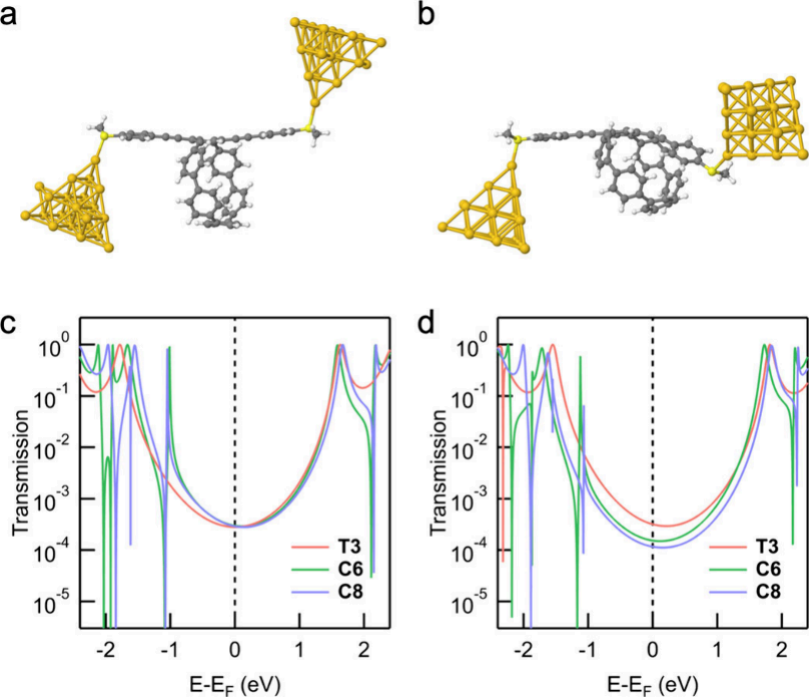
Junction geometries of **C6** optimized by DFT without (a) and with (b) vdW correction. DFT-based transmission functions calculated for **T3**, **C6**, and **C8** from the geometries optimized without (c) and with (d) vdW correction.

We then include the vdW correction based on Tkatchenko–Scheffler model and repeat all the calculations.^42^ The junction geometry of **C6** optimized with vdW correction exhibits a clearly noncoplanar backbone structure, as shown in Figure 3b. The phenylene group on one side is attracted to the CPP ring, causing it to rotate. Additionally, the connection bonds between this phenylene group and the central one are bent, further reducing orbital overlap, resulting in a lower conductance. The updated transmission functions are shown in Figure 3d. The values of **C6** and **C8** at the Fermi level are lower than that of **T3**, which is consistent with both tight-binding results and experiments.

In conclusion, our work has shown that integrating radially conjugated CPP units with linearly conjugated backbones influences single-molecule conductance significantly. The CPP-substituted molecules have conductance values roughly 1 order of magnitude lower than the phenyl-based analogs, owing to intramolecular π-interactions that induce nonplanarity and diminish π-orbital overlap along the conduction pathway. Both tight-binding models and DFT calculations consistently reveal that the distortions on the molecular backbone are critical in modulating electron transport. These findings not only deepen our understanding of the interplay between radial and linear conjugation in molecular junctions but also offer valuable insights for the future design of conjugated electronic materials bearing both of these conjugation topologies.

## Supplementary Material



## Notes

Synthesis details, molecular characterization data, NMR spectra, additional STM-BJ measurements, and tight-binding and DFT calculation methods (PDF)
