# Supplementary material for: Single-Molecule Conductance through Hybrid Radially and Linearly π‑Conjugated Macromolecules Reveals an Unusual Intramolecular π‑Interaction
Source: Nano Lett. 2025 Jul 24;25(31):12101–6. doi: 10.1021/acs.nanolett.5c03693 (PMC12333404; doi:10.1021/acs.nanolett.5c03693)
Supplement: Supplementary file 1 [file nl5c03693_si_001.pdf]

Supporting Information for:
Single-Molecule Conductance through Hybrid Radially and Linearly π-Conjugated Macromolecules Reveals an Unusual Intramolecular π-Interaction

Wanzhuo Shi[1,2†], Mengjiao Wang[3†], Latha Venkataraman[1,2,4]*, John D. Tovar[3,5]*

[†]equal contribution

[1]Department of Chemistry, Columbia University, New York, NY 10027, United States
[2]Institute of Science and Technology Austria, 3400 Klosterneuberg, Austria
[3]Department of Chemistry, Johns Hopkins University, Baltimore, MD 21218, United States
[4]Department of Applied Physics and Applied Mathematics, Columbia University, New York, NY 10027, United States
[5]Department of Materials Science and Engineering, Johns Hopkins University, Baltimore, MD 21218, United States

Contents

1. Synthesis 2
2. NMR spectra 7
3. Additional Conductance Measurements 13
4. Tight-binding model 16
5. DFT calculations 17
6. References 18

1. Synthesis

General Information

Moisture- and oxygen-sensitive reactions were carried out in flame-dried glassware and under an inert atmosphere of purified nitrogen using the syringe/septa technique or standard Schlenk procedures. Compound **1** (dialkynyl-[6]CPP), **2** (dialkynyl-[8]CPP), **3** (dialkynyl-terphenyl model),[1] **4**,[2] and **5**[3] were prepared according to the literature. Toluene was purified using an Innovative Technologies SPS-400-6 Solvent Purification System and further dried over Acros Organics 4Å molecular sieves. Diisopropylamine (D*i*PA) was dried over Acros Organics 4Å molecular sieves before use. All other solvents and reagents were purchased from Strem, Sigma-Aldrich, Oakwood Chemicals, or Ambeed and used without further purification.

^{1}H NMR spectra were obtained on either a Varian Inova 800 MHz Spectrometer or a Bruker Avance III HD 400 MHz Spectrometer, with residual protio-solvent resonances used as the internal standard ($CDCl_3$: 7.26 ppm, CD_2Cl_2: 5.32 ppm, toluene-d_8: 7.09, 7.01, 6.97, 2.08 ppm). Data are reported as: Chemical shift (multiplicity, integration, coupling constant). ^{13}C NMR spectra were obtained at 200 MHz on a Varian Inova 800 MHz Spectrometer, with solvent resonances as the internal standard (CD_2Cl_2: 53.84 ppm). Data are reported as chemical shift (ppm). Matrix-assisted laser desorption/ionization spectrometry (MALDI) was performed on a Bruker AutoFlex Max MALDI-TOF/TOF Mass Spectrometer using 1,8-dihydroxyanthracen-9(10*H*)-one (dithranol) as the matrix. Flash Chromatography was performed under manual air pressure on silica (SiO_2, 40-63 µm, 230-400 mesh). Thin Layer Chromatography (TLC) was performed using Analtech Silica Gel HLF TLC plates. Developed plates were visualized using UV light at 254 and 365 nm wavelengths.

Experimental details

C6

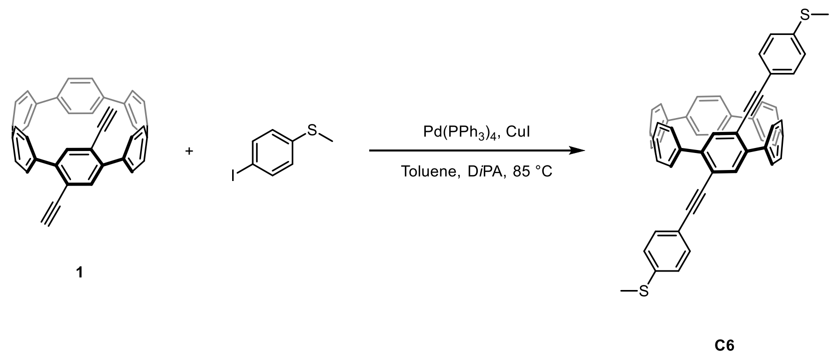

Dialkynyl-[6]CPP **1** (9.0 mg, 18 µmol, 1.0 eq) in a scintillation vial was dissolved in minimal dichloromethane and transferred into a flame-dried 25 mL Schlenk flask. The solvent was removed under reduced pressure by rotary evaporation, and the flask was held under vacuum (< 100 mTorr) for 2 h. (4-Iodophenyl)(methyl)sulfane (9.8 mg, 39 µmol, 2.2 eq), $Pd(PPh_3)_4$ (1.0 mg, 0.89 µmol, 0.050 eq), and copper(I) iodide (1.7 mg, 8.9 µmol, 0.50 eq) were added to the flask, which was then evacuated and backfilled with nitrogen for 5 cycles. Dry toluene (4 mL) and dry diisopropylamine (4 mL) were added via syringe. The reaction mixture was heated to 85 °C in an oil bath and was stirred for 16 h before being cooled

to room temperature. The reaction mixture was diluted with diethyl ether (10 mL). The organic layer was separated and washed with saturated NH_4Cl (aq.) (2 × 10 mL), brine (1 × 10 mL), dried over $MgSO_4$, and concentrated under reduced pressure. The crude material was purified by running a pipet column (silica, 20% dichloromethane in hexanes), yielding **C6** as a red solid (4.0 mg, 5.3 µmol, 30%).

^{1}H NMR (800 MHz, CD_2Cl_2) δ 8.42 (dd, 2H, *J* = 9.3, 2.0 Hz), 7.70 (s, 2H), 7.62 -7.56 (m, 12H), 7.54 - 7.51 (m, 8H), 7.49 (dd, 2H, *J* = 9.0, 2.1 Hz), 7.30 (d, 4H, *J* = 8.3 Hz), 2.55 (s, 6H).

^{13}C NMR (200 MHz, CD_2Cl_2) δ 140.73, 138.18, 136.58, 136.25, 135.99, 135.13, 134.53, 132.04, 132.00, 129.48, 129.00, 128.43, 128.12, 127.94, 127.73, 127.61, 126.22, 120.59, 119.70, 96.36, 90.06, 15.49.

MALDI (m/z): $[M]^+$ calculated for $C_{54}H_{36}S_2$, 748.226; found 748.086.

C8

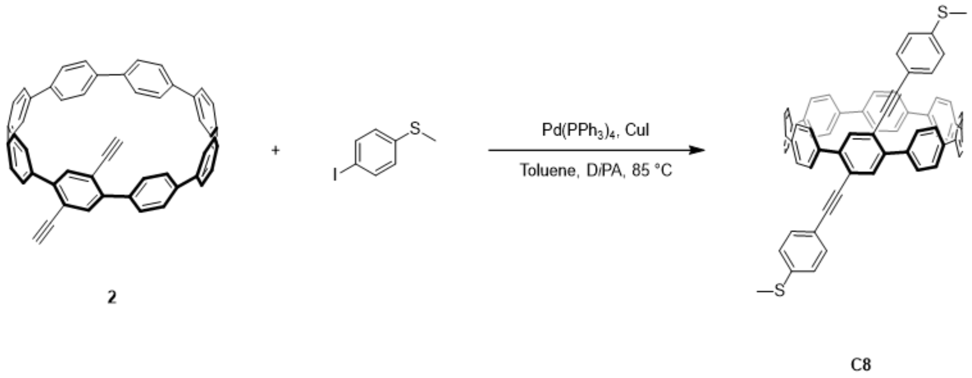

Dialkynyl-[8]CPP **2** (17 mg, 26 µmol, 1.0 eq) in a scintillation vial was dissolved in minimal dichloromethane and transferred into a flame-dried 25 mL Schlenk flask. The solvent was removed under reduced pressure by rotary evaporation, and the flask was held under vacuum (< 100 mTorr) for 2 h. (4-Iodophenyl)(methyl)sulfane (15 mg, 58 µmol, 2.2 eq), $Pd(PPh_3)_4$ (1.5 mg, 1.3 µmol, 0.050 eq), and copper(I) iodide (2.5 mg, 13 µmol, 0.50 eq) were added to the flask, which was then evacuated and backfilled with nitrogen for 5 cycles. Dry toluene (6 mL) and dry diisopropylamine (6 mL) were added via syringe. The reaction mixture was heated to 85 °C in an oil bath and was stirred for 20 h before being cooled to room temperature. The reaction mixture was diluted with diethyl ether (10 mL). The organic layer was separated and washed with saturated NH_4Cl (aq.) (2 × 10 mL), brine (1 × 10 mL), dried over $MgSO_4$, and concentrated under reduced pressure. The crude material was purified by running a flash column (silica, 20% dichloromethane in hexanes), yielding **C8** as a yellow solid (13 mg, 15 µmol, 58%).

^{1}H NMR (800 MHz, CD_2Cl_2) δ 7.74 (d, 4H, *J* = 6.7 Hz), 7.56 – 7.53 (m, 10H), 7.51 – 7.48 (m, 12H), 7.47 (s, 4H), 7.41 (d, 4H, *J* = 8.4 Hz), 7.25 (d, 4H, *J* = 8.3 Hz), 2.53 (s, 6H).

^{13}C NMR (200 MHz, CD_2Cl_2) δ 140.71, 140.55, 139.67, 138.41, 138.16, 138.03, 137.78, 137.22, 135.14, 135.08, 131.97, 131.94, 130.19, 128.26, 127.87, 127.77, 127.49, 127.45, 126.12, 121.57, 119.62, 94.60, 89.75, 15.48.

MALDI (m/z): $[M]^+$ calculated for $C_{66}H_{44}S_2$, 900.288; found 900.082.

T3

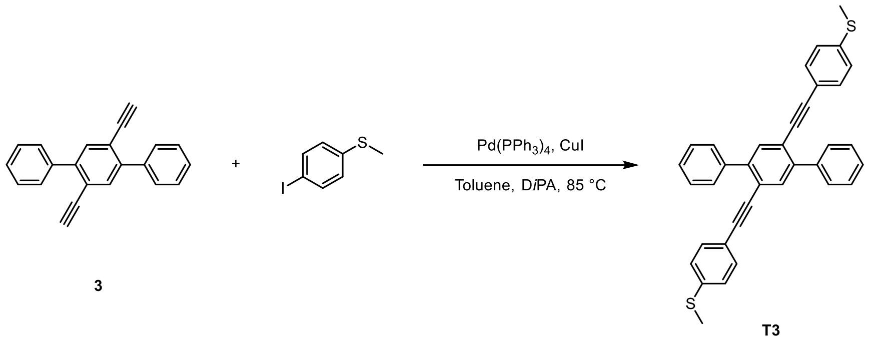

3

T3

A flame-dried 50 mL Schlenk flask was charged with dialkynylated terphenyl **3** (124 mg, 0.445 mmol, 1.00 eq), (4-iodophenyl)(methyl)sulfane (234 mg, 0.935 mmol, 2.10 eq), $Pd(PPh_3)_4$ (25.7 mg, 22.3 µmol, 0.0500 eq), and copper(I) iodide (8.5 mg, 45 µmol, 0.10 eq). The flask was evacuated and backfilled with nitrogen for 5 cycles. Dry toluene (10 mL) and diisopropylamine (10 mL) were added to the flask using syringes. The reaction mixture was heated to 85 °C in an oil bath and was stirred for 20 h before being cooled to room temperature. The reaction mixture was diluted with dichloromethane and saturated NH_4Cl (aq.) to form an off-white precipitate. The precipitate was collected after filtration and washed with DI H_2O and dichloromethane, yielding the desired **T3** as an off-white solid (69 mg, 0.13 mmol, 29%).

^{1}H NMR (400 MHz, toluene-*d*$_8$) δ 7.79 (s, 2H), 7.65 (d, 4H, *J* = 7.8 Hz), 7.27 (t, 4H, *J* = 7.6 Hz), 7.22 (d, 2H, *J* = 7.0 Hz), 7.19 (d, 4H, *J* = 8.2 Hz), 6.81 (d, 4H, *J* = 7.7 Hz), 1.85 (s, 6H).

The acquisition of **^{13}C NMR** was precluded due to the poor solubility of **T3**.

MALDI (m/z): $[M]^+$ calculated for $C_{36}H_{26}S_2$, 522.148; found 522.059.

6

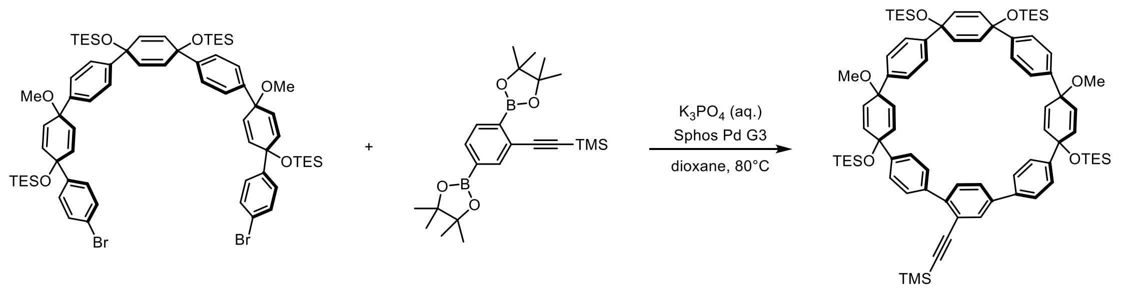

4 5 6

A flame-dried 100 mL Schlenk flask was charged with dibromo **4** (244 mg, 191 µmol, 1.00 eq), bisboronate **5** (97.6 mg, 229 µmol, 1.20 eq), and SPhos Pd GIII (15 mg, 19 µmol, 0.10 eq). The flask was evacuated and backfilled with nitrogen for 5 cycles. Dry 1,4-dioxane (40 mL) was added to the flask using a syringe. The reaction mixture was heated to 85 °C in an oil bath before an aqueous solution of K_3PO_4 (2 M, 4 mL) was added. The reaction was stirred at 85 °C for 20 h before being cooled to room temperature. The 1,4-dioxane was removed under reduced pressure, and the resulting material was filtered through a celite pad with DI H_2O and dichloromethane. The organic layer was separated, dried over $MgSO_4$, and

concentrated under reduced pressure. The crude material was purified by running a flash column (silica, 0% to 2% ethyl acetate in hexanes), yielding the desired macrocycle **6** as a yellow solid (47 mg, 36 µmol, 18%).

^{1}H NMR (400 MHz, CD_2Cl_2) δ7.76 (s, 1H), 7.49 - 7.45 (m, 3H), 7.39 (d, 2H, *J* = 8.3 Hz), 7.35 – 7.25 (m, 9H), 7.12 (d, 2H, *J* = 8.1 Hz), 7.05 (d, 2H, *J* = 8.2 Hz), 6.29 (d, 2H, *J* = 6.6 Hz), 6.26 (d, 2H, J = 6.9 Hz), 6.14 (s, 4H), 6.00 (d, 2H, *J* = 10.0 Hz), 5.94 (d, 2H, *J* = 9.8 Hz), 3.32 (s, 3H), 3.31 (s, 3H), 1.05 – 1.01 (m, 18H), 0.91 – 0.87 (m, 18H), 0.76 – 0.70 (m, 12H), 0.55 – 0.48 (m, 12H), 0.18 (s, 9H).

^{13}C NMR (100 MHz, CD_2Cl_2) δ 146.99, 146.93, 144.91, 144.81, 142.44, 142.28, 139.56, 139.46, 136.50, 136.14, 134.12, 132.02, 131.17, 129.87, 129.57, 129.33, 128.84, 127.57, 126.82, 126.66, 126.28, 125.70, 105.19, 97.91, 75.07, 74.95, 72.79, 72.49, 69.97, 69.92, 52.12, 7.39, 7.27, 7.05, 6.91, 0.17.

MALDI (m/z): $[M-CH_2O]^+$ calculated for $C_{78}H_{104}O_5Si_5$, 1260.673; found 1260.349.

7

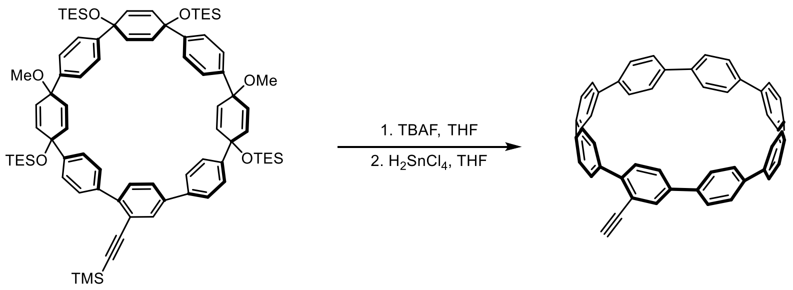

6 7

Macrocycle 6 (47 mg, 36 µmol, 1.0 eq) was dissolved in 1 mL THF, and a 1M solution of TBAF in THF (0.29 mL, 0.29 mmol, 8.0 eq) was added dropwise. The reaction was stirred at room temperature under nitrogen for 1 h, and a white precipitate was formed after the reaction was quenched with 10 mL DI H_2O. The precipitate was collected after filtration and washed with DI H_2O and dichloromethane. The collected white solid was dissolved in dissolved in minimal acetone and transferred into a flame-dried 25 mL Schlenk flask. The solvent was removed under reduced pressure by rotary evaporation, and the flask was held under vacuum (< 100 mTorr) for 2 h. The flask was evacuated and backfilled with nitrogen for 3 cycles. A solution of $SnCl_2 \cdot 2H_2O$ (27 mg, 0.12 mmol, 3.3 eq) and 12 M HCl (20 µL, 0.24 mmol, 6.6 eq) in THF (0.90 mL) was added dropwise. The reaction was stirred at room temperature under nitrogen for 1 h before being quenched with a 1 M aqueous solution of NaOH. The reaction mixture was extracted with dichloromethane (3 × 5 mL). The combined organic layer was dried over $MgSO_4$ and concentrated under reduced pressure. The crude material was purified by running a pipet column (silica, 20% dichloromethane in hexanes), yielding the alkynylated [8]CPP **7** as a yellow solid (14 mg, 23 µmol, 63% after 2 steps).

^{1}H NMR (400 MHz, $CDCl_3$) δ 7.87 (d, 1H, *J* = 2.1 Hz), 7.69 (d, 2H, *J* = 8.9 Hz), 7.54 – 7.41 (m, 26H), 7.10 (dd, 1H. *J* = 8.8, 2.0 Hz),6.97 (d, 1H, *J* = 8.7 Hz), 3.26 (s, 1H).

^{13}C NMR (100 MHz, $CDCl_3$) δ 141.14, 139.43, 138.25, 138.14, 137.99, 137.94, 137.88, 137.86, 137.84, 137.71, 137.47, 137.36, 132.64, 130.76, 130.64, 128.84, 128.10, 127.84, 127.64, 127.56, 118.42, 83.73, 80.36.

MALDI (m/z): $[M]^+$ calculated for $C_{50}H_{32}$, 632.250; found 631.837.

Half-C8

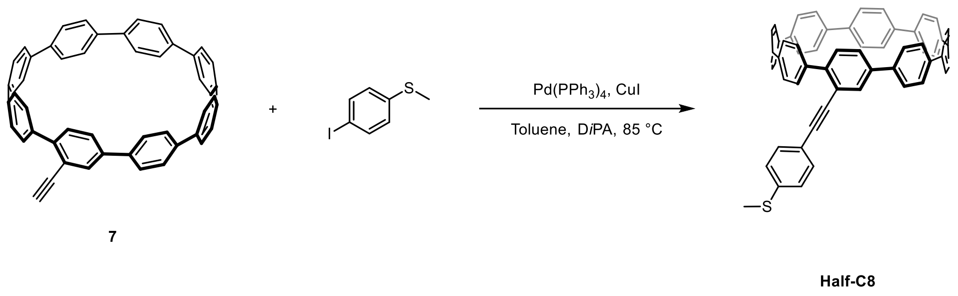

Alkynylated [8]CPP **7** (14 mg, 23 µmol, 1.0 eq) in a scintillation vial was dissolved in minimal dichloromethane and transferred into a flame-dried 25 mL Schlenk flask. The solvent was removed under reduced pressure by rotary evaporation, and the flask was held under vacuum (< 100 mTorr) for 2 h. (4-Iodophenyl)(methyl)sulfane (6.8 mg, 27 µmol, 1.2 eq), $Pd(PPh_3)_4$ (1.3 mg, 1.1 µmol, 0.050 eq), and copper(I) iodide (2.2 mg, 11 µmol, 0.50 eq) were added to the flask, which was then evacuated and backfilled with nitrogen for 5 cycles. Dry toluene (5 mL) and dry diisopropylamine (5 mL) were added via syringe. The reaction mixture was heated to 85 °C in an oil bath and was stirred for 18 h before being cooled to room temperature. The reaction mixture was diluted with diethyl ether (10 mL). The organic layer was separated and washed with saturated NH_4Cl (aq.) (2 × 10 mL), brine (1 × 10 mL), dried over $MgSO_4$, and concentrated under reduced pressure. The crude material was purified by running a pipet column (silica, 20% dichloromethane in hexanes), yielding **Half-C8** as a yellow solid (5.4 mg, 7.2 µmol, 32%).

^{1}H NMR (400 MHz, $CDCl_3$) δ 7.87 (d, 1H, J = 2.0 Hz), 7.75 (d, 2H, J = 8.8 Hz), 7.53 – 7.41 (m, 28H), 7.21 (d, 2H, J = 8.4 Hz), 7.10 (dd, 1H, J = 8.6, 2.1 Hz), 7.02 (d, 1H, J = 8.6 Hz), 2.51 (s, 3H).

^{13}C NMR (150 MHz, CD_2Cl_2) δ 140.54, 140.28, 139.48, 138.54, 138.35, 138.21, 138.09, 138.00, 137.82, 137.66, 132.70, 132.10, 130.27, 130.16, 129.00, 127.83, 126.12, 120.03, 119.76, 92.51, 89.95, 15.47.

MALDI (m/z): $[M]^+$ calculated for $C_{57}H_{38}S$, 754.269; found 754.137.

2. NMR spectra

C6

^{1}H (top, 800 MHz) and ^{13}C (bottom, 200 MHz) NMR in CD_2Cl_2

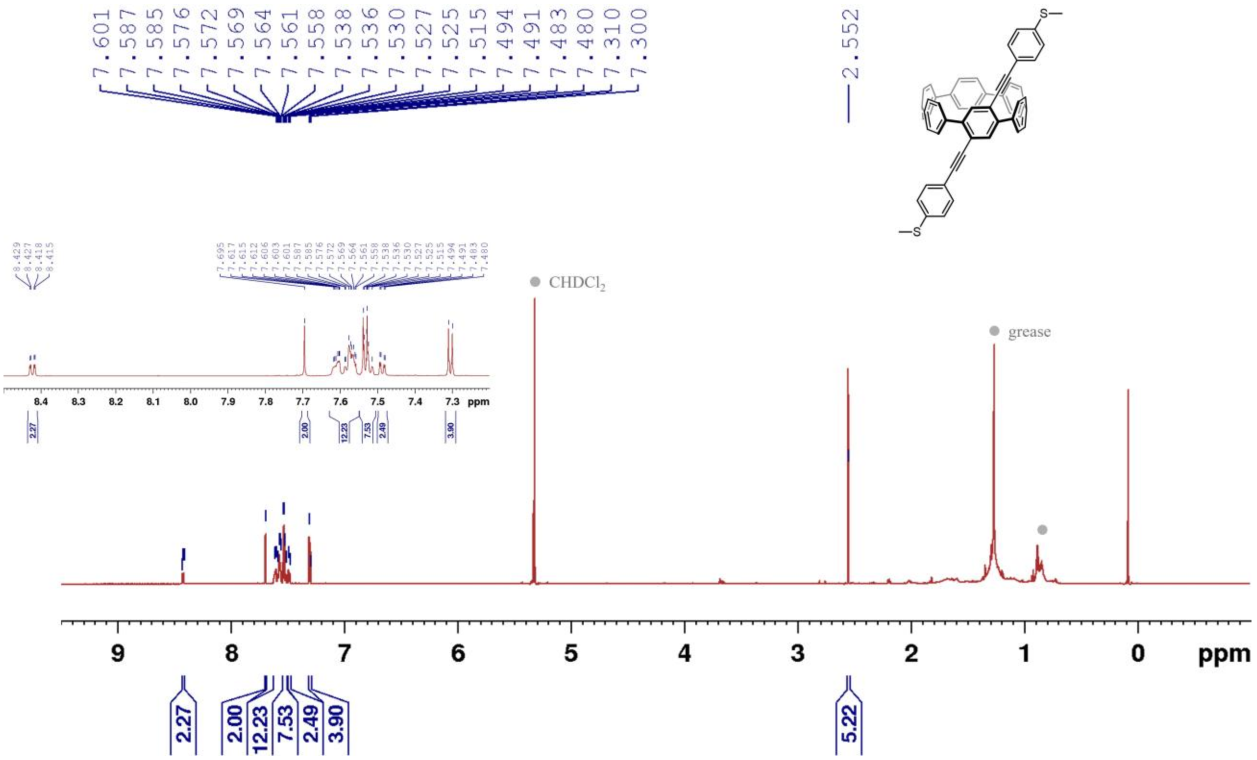

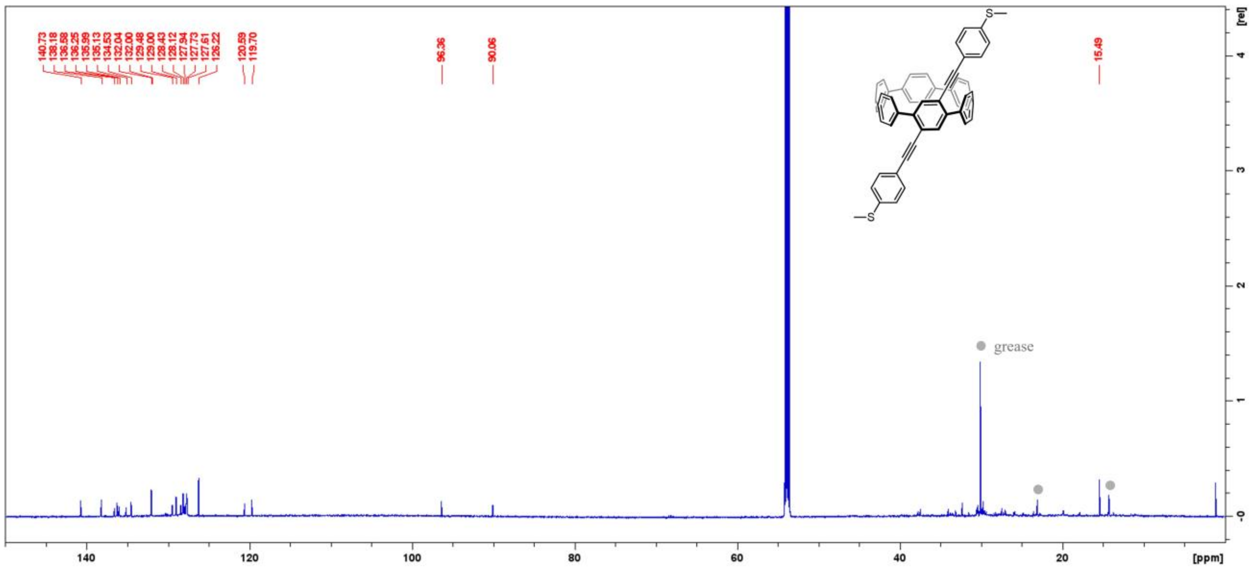

C8

^{1}H (top, 800 MHz) and ^{13}C (bottom, 200 MHz) NMR in CD_2Cl_2

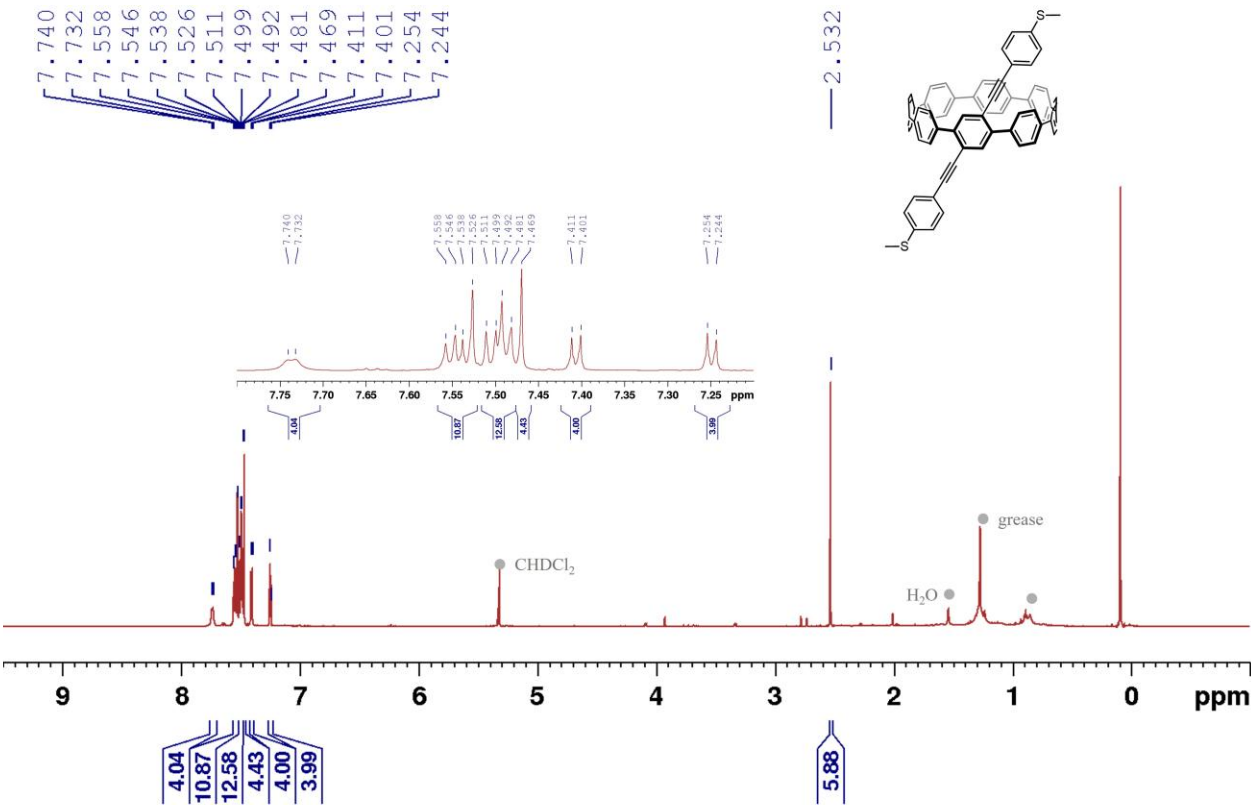

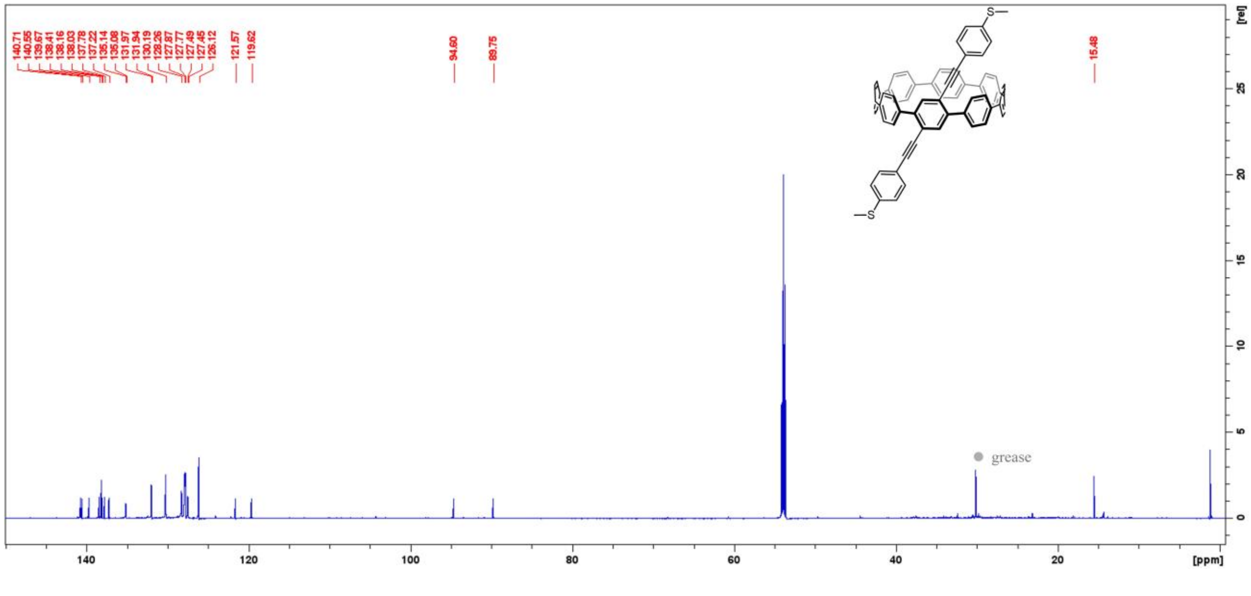

T3

^{1}H (400 MHz) NMR in toluene-d_8

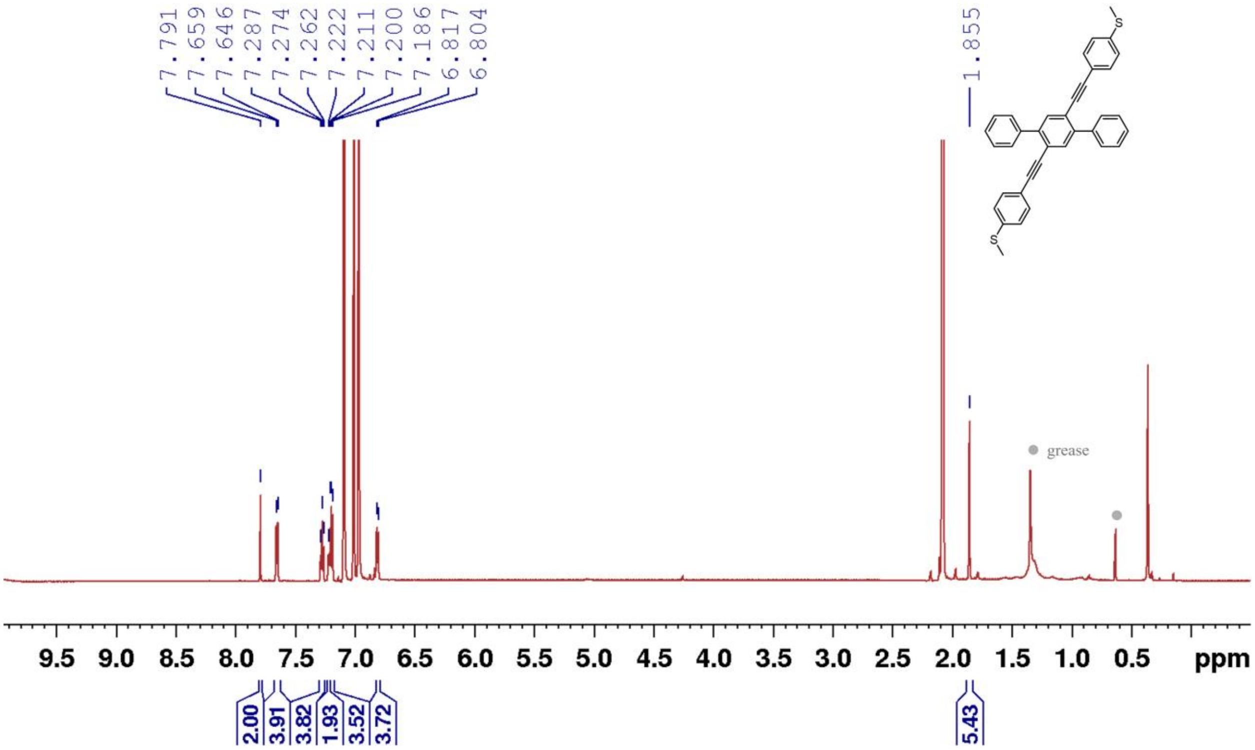

6

^{1}H (top, 400 MHz)and ^{13}C (bottom, 100 MHz) NMR in CD_2Cl_2

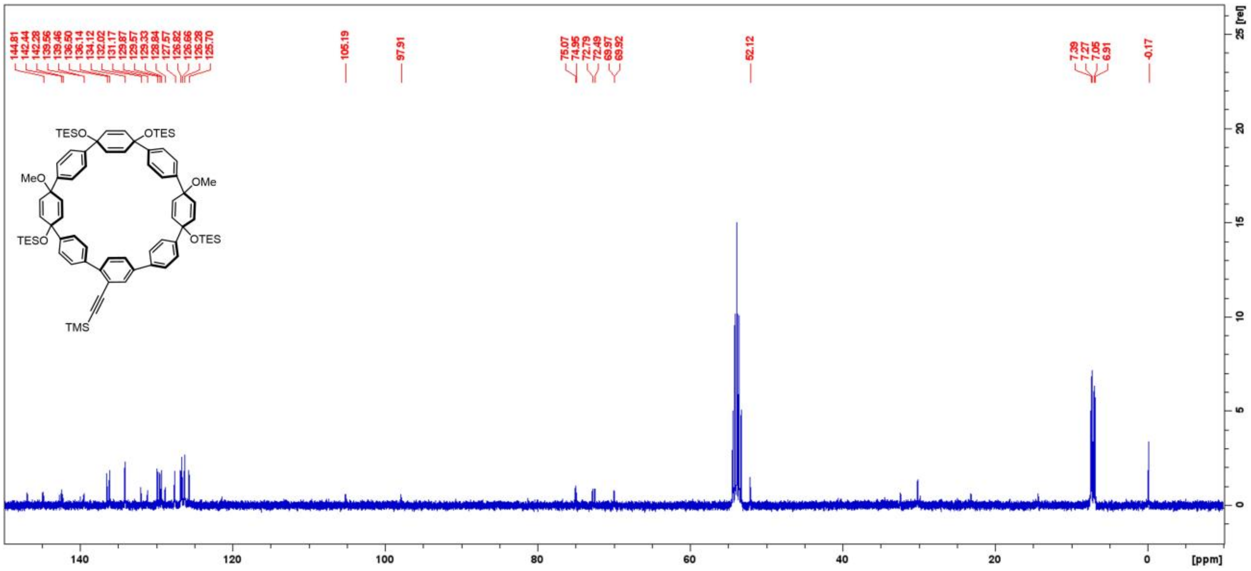

7

<u>^{1}H (top, 400 MHz) and ^{13}C (bottom, 100 MHz) NMR in $CDCl_3$</u>

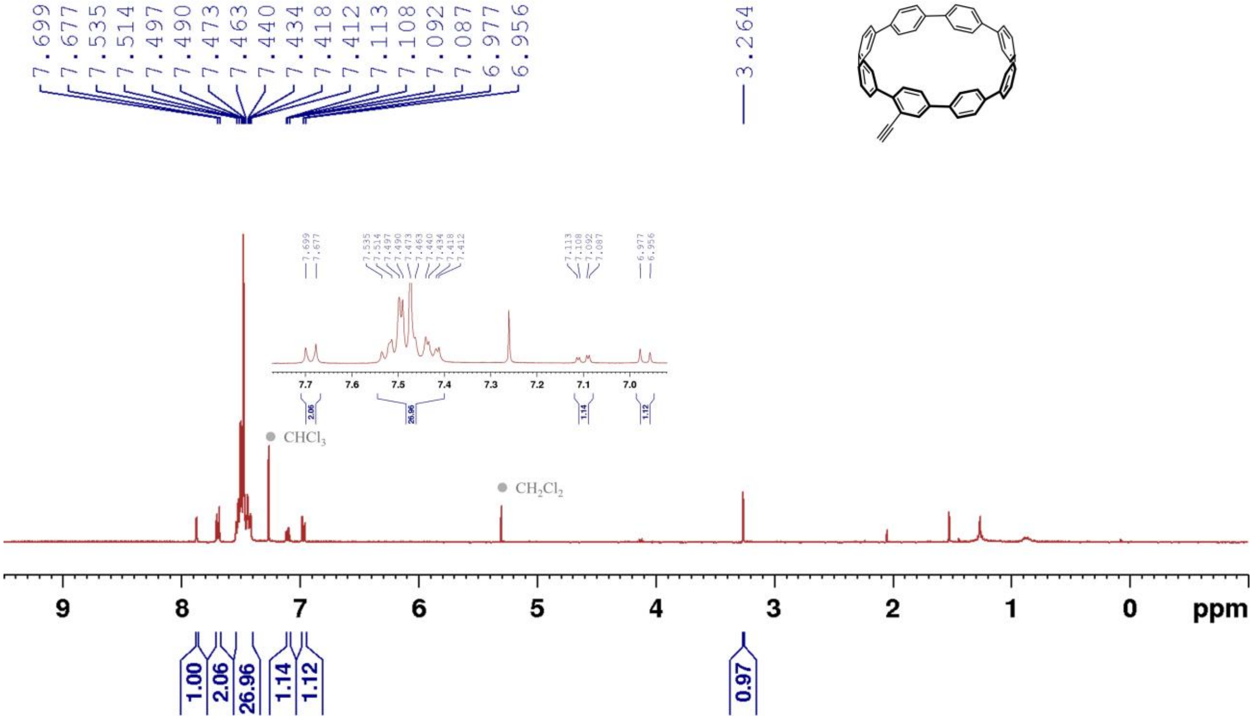

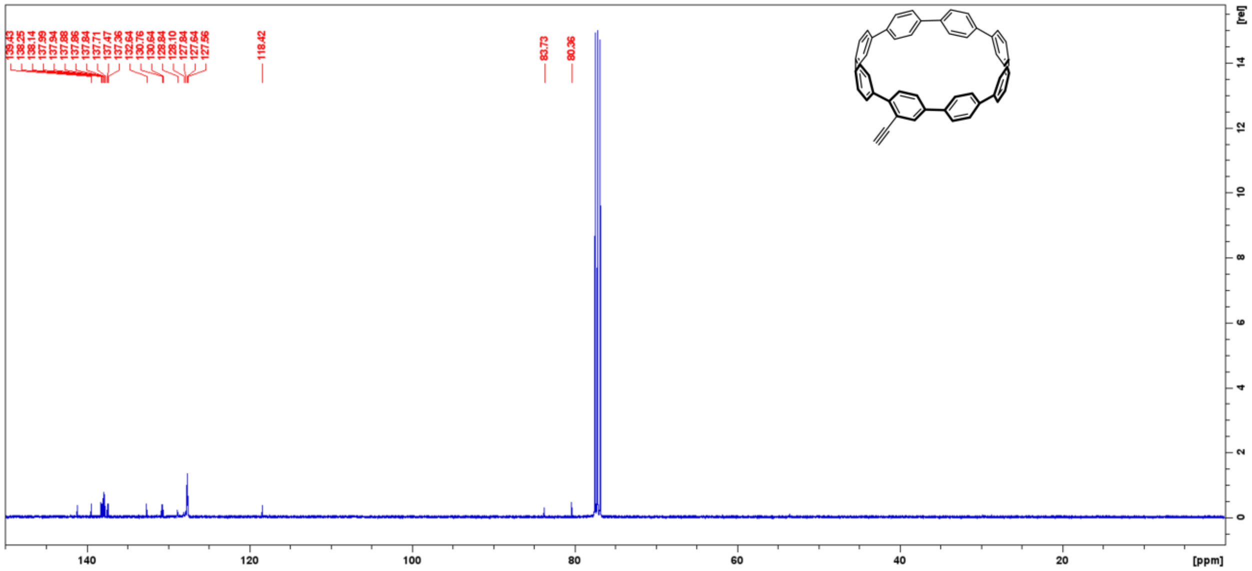

Half-C8

^{1}H (top, 400 MHz) NMR in $CDCl_3$ and ^{13}C (bottom, 150 MHz) NMR in CD_2Cl_2

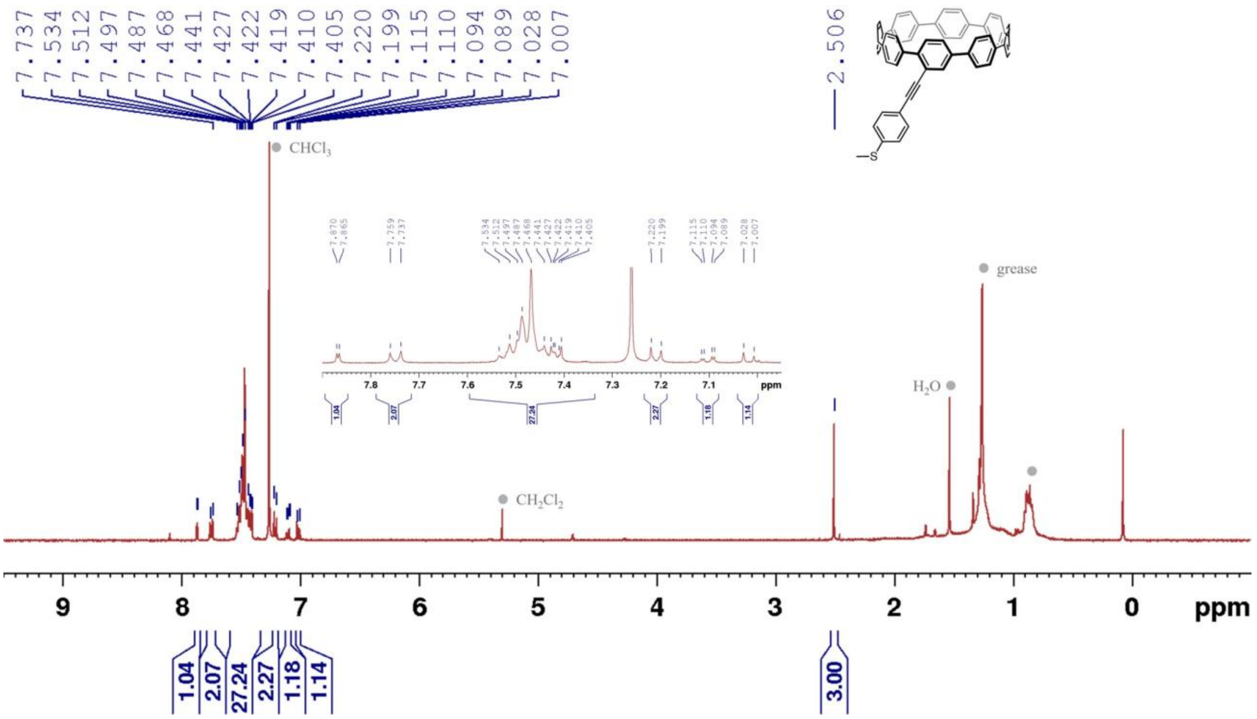

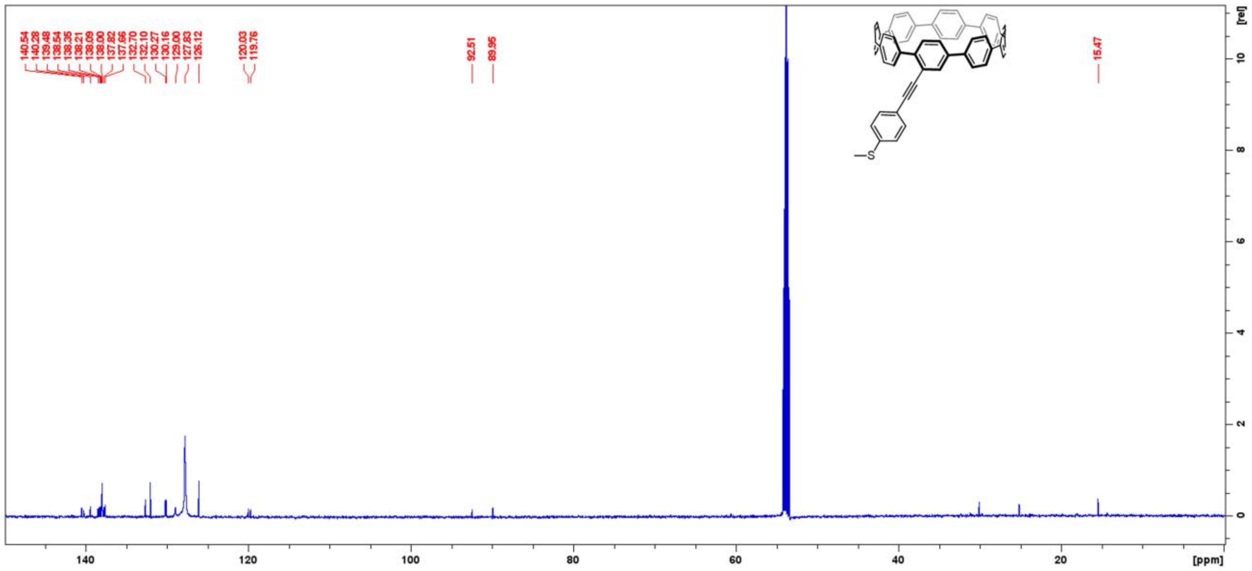

3. Additional Conductance Measurements

Conductance measurements in Propylene Carbonate (PC)

Conductance measurements for **T3**, **C6** and **C8** were also carried out in 100 μM propylene carbonate (PC) solutions. Except for the change in solvent, all other experimental settings were identical to those used for the TCB data presented in Figure 1 of the main text. The resulting one-dimensional (a) and two-dimensional (b, c, d) histograms are shown in Figure S1.

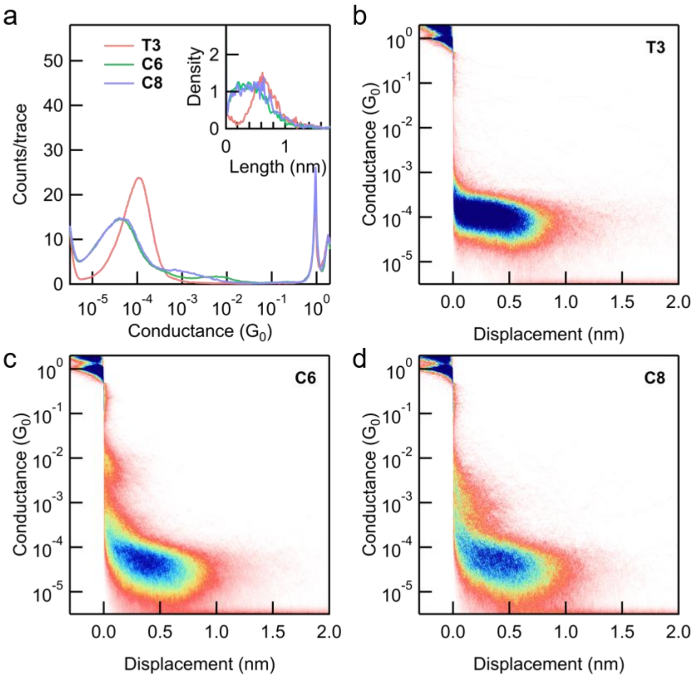

*Figure S1. (a) Logarithmically binned 1D conductance histograms for **T3**, **C6** and **C8** measured in PC solutions. Each histogram is generated from over 3000 traces without selection. Inset: normalized molecular plateau length distribution histograms. The corresponding 2D conductance-displacement histograms for **T3** (b), **C6** (c) and **C8** (d) are shown.*

We obtain the most probable conductance by fitting the peaks with Gaussian functions. The resulting conductances are $2.3 \times 10^{-4}\ G_0$ for **T3** and $3.9 \times 10^{-5}\ G_0$ for both **C6** and **C8**. Although these values differ from those measured in TCB, this discrepancy is expected, since solvents are known to affect the surface Fermi level alignment and therefore modify conductance.[4-7] Notably, the same trend is observed as in the TCB experiments: the **T3** molecule without the CPP group exhibits higher conductance than those with CPP cycles (**C6** and **C8**). Furthermore, the distribution of plateau step lengths indicates that **T3** exhibits a higher probability of longer junctions compared to **C6** and **C8** in PC, reinforcing the conclusion that the bulky CPP cycle on the side obstructs single-molecule junction formation. We further note that, in PC, the difference in plateau step length between phenyl-substitute (**T3**) and CPP-substitutes (**C6** and **C8**) is smaller than in TCB, suggesting that CPP's obstruction on forming Au-SMe contacts is less in PC. The decreased peaks of the Au-CPP-Au junctions in PC supports this observation.

Conductance measurements of Half-C8

We performed conductance measurements for **Half-C8** (structure shown in Figure S2a) at a 100 mV tip bias in 100 μM TCB solution. The associated 1D and 2D histograms are shown in Figures S2b and S2c, respectively. Notably, the main conductance peak at $2.7 \times 10^{-5} G_0$ is absent, which supports that the junctions are formed by SMe linker groups on both sides, allowing electrons to tunnel through the entire aryl alkyne-based backbone.

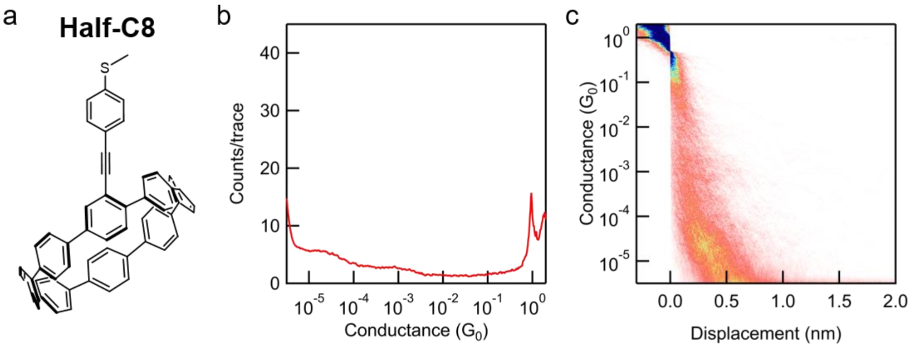

*Figure S2. (a) Structure of **Half-C8** analog. (b, c) Logarithmically binned 1D and 2D conductance histograms for **Half-C8** measured in TCB. Histograms are generated from 3000 traces without selection.*

Conductance measurements of [6]CPP

We performed conductance measurements for **[6]CPP** (structure shown in Figure S3a) at a 100 mV tip bias in 100 μM TCB solution. The associated 1D and 2D histograms are shown in Figures S3b and S3c, respectively. We observe a distinct, broad conductance peak around $10^{-2}\ G_0$, consistent with previous studies.[8, 9] This peak aligns with the faint one seen in **C6**, suggesting that it originates from the formation of Au-CPP-Au junctions. The lower prominence of this peak for **C6** implies that the SMe groups are competing for binding to the Au electrode surfaces, forming junctions with the conductance of $2.7 \times 10^{-5}\ G_0$.

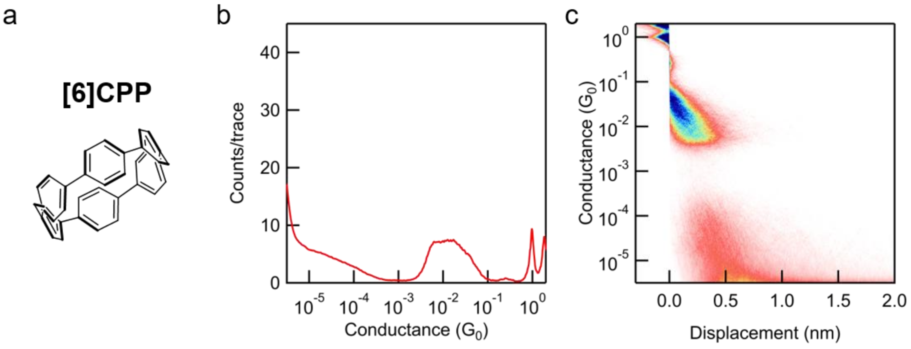

*Figure S3. (a) Structure of **[6]CPP**. (b, c) Logarithmically binned 1D and 2D conductance histograms for **Half-C8** measured in TCB. Histograms are generated from 3000 traces without selection.*

Noise analysis of C6 and [6]CPP

To validate that the high conductance peak indeed originates from the Au–CPP–Au junction, we analyzed the noise of traces. We first isolated the segment whose conductance falls inside the peak range, calculated the noise power spectral density (PSD). All three data sets, i.e., two peaks for **C6** and one for bare **[6]CPP**, display a $1/f^{1.6}$ frequency dependence, excluding setup artefacts.

Plotting the integrated noise power against the averaged conductance for thousands of individual junctions reveals distinct power-law regimes (Figure S4). In **C6**, the noise of high-G junctions scales with $G^{1.7}$, whereas the low-G junctions scales with $G^{1.0}$. Bare **[6]CPP** has an exponent of roughly 1.4. It has been well known that when the molecule is coupled to the metal electrodes through bond, the noise power scales linearly with conductance, while through space coupling pushes the exponent towards 2.[10] The linear scaling for the **C6** low-G peak therefore is confirmed to a junction anchored via thiomethyl groups, whereas the 1.7 exponent of the high-G peak indicates a through space path in an Au–CPP–Au configuration. The 1.4 value for **[6]CPP** is consistent with its better exposed π-system that could couple to the electrodes stronger by face-to-face overlap and less sensitive to the interface structural fluctuation, agreeing with its slightly higher conductance and longer plateau length.

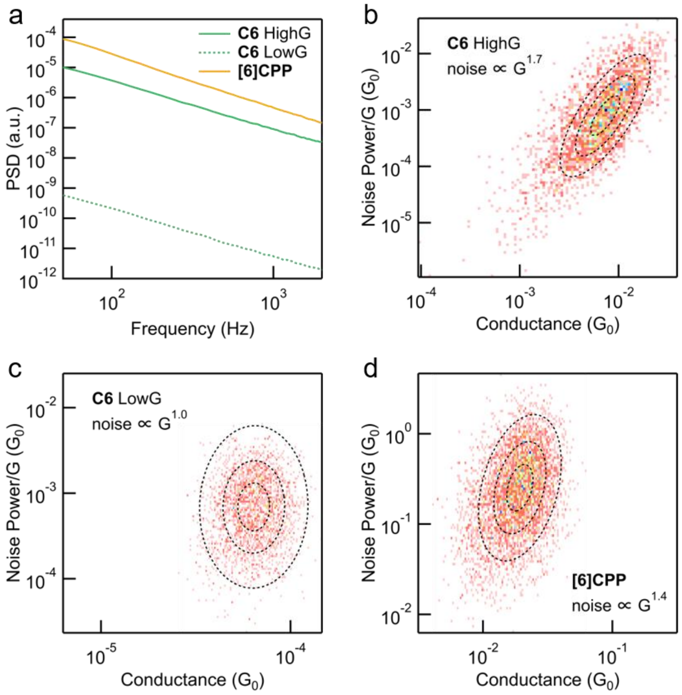

Figure S4. (a) Average power spectral densities (PSDs) extracted from conductance plateaus: ***C6*** *high-G (solid green),* ***C6*** *low-G (dashed green) and [6]CPP (orange). (b-d) Two-dimensional histograms of integrated noise power versus mean conductance for the high-G and low-G plateaus of* ***C6*** *and [6]CPP, respectively.*

4. Tight-binding model

In a single-molecule junction model, the contacts between the molecule and the electrodes prevents it from being considered as an isolated system. Consequently, the Hamiltonian H' should be modeled as the sum of the isolated molecule's Hamiltonian (H_{mol}) and the interaction terms between the molecule and the electrodes, as follows:[11]

$$H' = H_{mol} + \Sigma_L + \Sigma_R \qquad S1$$

where Σ_L and Σ_R are the self-energy terms of the left and right electrodes, respectively. The couplings ($\Gamma_{L,R}$) between the molecule and the electrodes are described by the anti-Hermitian part of the self-energies:[11]

$$\Gamma_{L,R} = i\left(\Sigma_{L,R} - \Sigma_{L,R}^{\dagger}\right) = -2\mathrm{Im}\left[\Sigma_{L,R}\right] \qquad S2$$

After establishing the tight-binding Hamiltonians and the coupling terms, we use Eqs. (1) and (2) from the main text to calculate the model's transmission functions. In practice, we construct H_{mol} using the Hückel model, setting the on-site energies of all carbon atoms in the backbone to zero and representing the C–C bonds with a hopping integral. The SMe groups are omitted and accounted for in the system via the coupling $\Gamma_{L,R}$.

The Mathematica code employed is shown below.

```
(*  Define the system Hamiltonian as a function of nCPP  *)
t=-1.0; (*  Hopping integral  *)
sCPP = 1; (*  Scaling factor on the bonds between phenyls of the CPP cycle  *)
sBackbone = 1; (* Scaling factor on the backbone *)

Hmol[nCPP_]:=Module[{nCarbon=6*nCPP+16,h},
  h=ConstantArray[0,{nCarbon,nCarbon}]; (* Initialize zero matrix *)

  (* CPP couplings *)
  For[i=0,i<nCPP,i++,
   For[j=1,j<6,j++,
    h[[6*i+j,6*i+j+1]]=t;
    h[[6*i+j+1,6*i+j]]=t];
   h[[6*i+1,6*i+6]]=t; (* Finish phenyl loop *)
   h[[6*i+6,6*i+1]]=t];
  For[i=0,i<(nCPP-1),i++,
   h[[6*i+4,6*i+7]]=t*sCPP;
   h[[6*i+7,6*i+4]]=t*sCPP];
  If[nCPP>1,h[[6*nCPP-2,1]]=t*sCPP;h[[1,6*nCPP-2]]=t*sCPP;];

  (* First aryl alkyne branch is connected to the 2nd atom on the CPP cycle *)
  h[[2,6*nCPP+1]]=0.8*t; (* Single bond *)
  h[[6*nCPP+1,2]]=0.8*t;
  h[[6*nCPP+1,6*nCPP+2]]=1.2*t; (* Triple bond *)
  h[[6*nCPP+2,6*nCPP+1]]=1.2*t;
  h[[6*nCPP+2,6*nCPP+3]]=0.8*t;
  h[[6*nCPP+3,6*nCPP+2]]=0.8*t;
  Do[Module[{base=6*nCPP+3},
```

```
  h[[base+i,base+i+1]]=t;
  h[[base+i+1,base+i]]=t],{i,0,4}];
 h[[6*nCPP+8,6*nCPP+3]]=t; (* Finish phenyl loop *)
 h[[6*nCPP+3,6*nCPP+8]]=t;

 (* Second aryl alkyne branch is connected to the 5th atom on the CPP cycle *)
 h[[5,6*nCPP+9]]=0.8*t; (* Single bond *)
 h[[6*nCPP+9,5]]=0.8*t;
 h[[6*nCPP+9,6*nCPP+10]]=1.2*t; (* Triple bond *)
 h[[6*nCPP+10,6*nCPP+9]]=1.2*t;
 h[[6*nCPP+10,6*nCPP+11]]=0.8*t*sBackbone;
 h[[6*nCPP+11,6*nCPP+10]]=0.8*t*sBackbone;
 Do[Module[{base=6*nCPP+11},
  h[[base+i,base+i+1]]=t;
  h[[base+i+1,base+i]]=t],{i,0,4}];
 h[[6*nCPP+16,6*nCPP+11]]=t; (* Finish phenyl loop *)
 h[[6*nCPP+11,6*nCPP+16]]=t;
 h]

(* Coupling between Au and molecule *)
GammaLR=0.1; (* Symmetric coupling *)
ΓL[nCPP_,siteL_]:=SparseArray[{{siteL,siteL}->GammaLR},{6*nCPP+16,6*nCPP+16}]
ΓR[nCPP_,siteR_]:=SparseArray[{{siteR,siteR}->GammaLR},{6*nCPP+16,6*nCPP+16}]

(* Define the retarded Green's function *)
Gret[En_,nCPP_,siteL_,siteR_]:=Inverse[En*IdentityMatrix[6*nCPP+16]-
Hmol[nCPP]+I/2*(ΓL[nCPP,siteL]+ΓR[nCPP,siteR])]

(* Calculate the transmission function *)
T[En_,nCPP_,siteL_,siteR_]:=Re[Tr[ΓL[nCPP,siteL].Gret[En,nCPP,siteL,siteR].ΓR[nCPP,siteR].
ConjugateTranspose[Gret[En,nCPP,siteL,siteR]]]]
```

5. DFT calculations

DFT calculations were performed using the FHI-AIMS package.[12, 13] For each molecule, the geometry of the molecule with one Au atom on each side was first relaxed, using the B3LYP functional.[14-16] Standard convergence criteria in the self-consistent field cycle were applied: an electron density difference of 10^{-5} electrons Å^{-3}, a total energy difference of 10^{-6} eV, a sum of Kohn–Sham eigenvalues difference of 10^{-4} eV, and a force difference of 10^{-4} eV Å^{-1}. Next, Au_{21} pyramids were then appended as shown in Figure S5 to simulate the molecular junction and used for transmission calculation. Finally, the energy-dependent electronic transmission was calculated with the AITRANSS package, which uses a non-equilibrium Green's function approach.[17-21]

We compare the geometries of the Au–molecule–Au junctions optimized both without (a–c) and with (d–f) vdW corrections based on the Tkatchenko–Scheffler model in Figure S5.[22] When the vdW correction is not included, the molecular backbones remain flat regardless of the side groups. However, adding the vdW correction induces a distinct difference between the phenyl-substituted **T3** and the CPP-substituted (**C6** and **C8**). For **T3**, including the vdW correction does not noticeably alter the geometry. In contrast, for

C6 and **C8**, the geometries optimized with vdW corrections are clearly bent: the CPP ring and one aryl acetylene branch move closer together, attracting and twisting the phenyl ring on the backbone. To quantify the structural distortion, we labeled the dihedral angle between the planes defined by the two phenyl rings on each aryl acetylene branch. This structural change indicates that intramolecular π-interactions distort the geometry of the backbone and reduce the π-orbital overlap along the conduction pathway, leading to lower conductance.

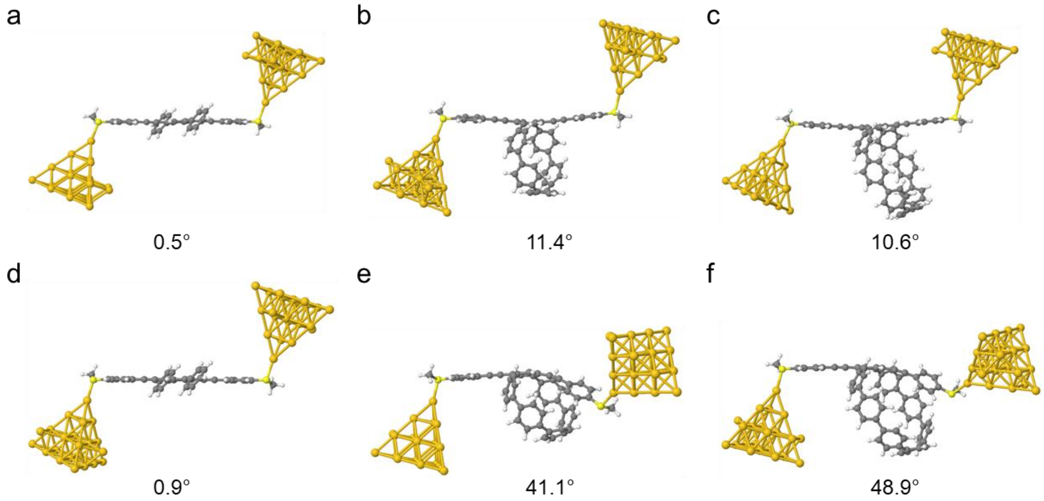

*Figure S5. Au–molecule–Au complexes of **T3**, **C6**, and **C8**. Panels (a–c) show the structures obtained without vdW corrections, and panels (d–f) show those obtained with vdW corrections. Dihedral angle between the planes of the two phenyl rings on each aryl acetylene branch is labeled.*

6. References

(1) Peters, G. M.; Grover, G.; Maust, R. L.; Colwell, C. E.; Bates, H.; Edgell, W. A.; Jasti, R.; Kertesz, M.; Tovar, J. D., Linear and Radial Conjugation in Extended pi-Electron Systems, *J. Am. Chem. Soc.,* **2020,** 142, 2293-2300.

(2) Lovell, T. C.; Colwell, C. E.; Zakharov, L. N.; Jasti, R., Symmetry breaking and the turn-on fluorescence of small, highly strained carbon nanohoops, *Chem. Sci.,* **2019,** 10, 3786-3790.

(3) Siddique, R. G.; Arachchige, K. S.; AL‐Fayaad, H. A.; Thoburn, J. D.; McMurtrie, J. C.; Clegg, J. K., Controlling the Complexity and Interconversion Mechanisms in Self‐Assembled [Fe2L3] 4+ Helicates and [Fe4L6] 8+ Cages, *Angew. Chem. Int. Ed.,* **2022,** 61, e202115555.

(4) Nakashima, S.; Takahashi, Y.; Kiguchi, M., Effect of the environment on the electrical conductance of the single benzene-1,4-diamine molecule junction, *Beilstein J. Nanotechnol.,* **2011,** 2, 755-759.

(5) Kotiuga, M.; Darancet, P.; Arroyo, C. R.; Venkataraman, L.; Neaton, J. B., Adsorption-Induced Solvent-Based Electrostatic Gating of Charge Transport through Molecular Junctions, *Nano Lett.,* **2015,** 15, 4498-4503.

(6) Dalmieda, J.; Shi, W.; Li, L.; Venkataraman, L., Solvent-Mediated Modulation of the Au-S Bond in Dithiol Molecular Junctions, *Nano Lett.,* **2024,** 24, 703-707.

(7) Shi, W.; Greenwald, J. E.; Venkataraman, L., Impact of Solvent Electrostatic Environment on Molecular Junctions Probed via Electrochemical Impedance Spectroscopy, *Nano Lett.,* **2024,** 24, 9283-9288.

(8) Lv, Y.; Lin, J.; Song, K.; Song, X.; Zang, H.; Zang, Y.; Zhu, D., Single cycloparaphenylene molecule devices: Achieving large conductance modulation via tuning radial pi-conjugation, *Sci. Adv.,* **2021,** 7, eabk3095.
(9) Lin, J.; Lv, Y.; Song, K.; Song, X.; Zang, H.; Du, P.; Zang, Y.; Zhu, D., Cleavage of non-polar C(sp(2))–C(sp(2)) bonds in cycloparaphenylenes via electric field-catalyzed electrophilic aromatic substitution, *Nat. Commun.,* **2023,** 14, 293.
(10) Adak, O.; Rosenthal, E.; Meisner, J.; Andrade, E. F.; Pasupathy, A. N.; Nuckolls, C.; Hybertsen, M. S.; Venkataraman, L., Flicker noise as a probe of electronic interaction at metal–single molecule interfaces, *Nano Lett.,* **2015,** 15, 4143-4149.
(11) Datta, S., *Electronic transport in mesoscopic systems*. Cambridge University Press: Cambridge, UK ; New York, 1997.
(12) Blum, V.; Gehrke, R.; Hanke, F.; Havu, P.; Havu, V.; Ren, X. G.; Reuter, K.; Scheffler, M., molecular simulations with numeric atom-centered orbitals, *Comput. Phys. Commun.,* **2009,** 180, 2175-2196.
(13) Ren, X. G.; Rinke, P.; Blum, V.; Wieferink, J.; Tkatchenko, A.; Sanfilippo, A.; Reuter, K.; Scheffler, M., Resolution-of-identity approach to Hartree-Fock, hybrid density functionals, RPA, MP2 and with numeric atom-centered orbital basis functions, *New Journal of Physics,* **2012,** 14, 053020.
(14) Becke, A. D., Density-functional thermochemistry. III. The role of exact exchange, *J. Chem. Phys.,* **1993,** 98, 5648-5652.
(15) Vosko, S. H.; Wilk, L.; Nusair, M., Accurate spin-dependent electron liquid correlation energies for local spin density calculations: a critical analysis, *Can. J. Phys.,* **1980,** 58, 1200-1211.
(16) Scuseria, G. E., Recent progress in the development of exchange-correlation functionals., *Abstracts of Papers of the American Chemical Society,* **2005,** 229, U763-U763.
(17) Arnold, A.; Weigend, F.; Evers, F., Quantum chemistry calculations for molecules coupled to reservoirs: Formalism, implementation, and application to benzenedithiol, *J. Chem. Phys.,* **2007,** 126.
(18) Wilhelm, J.; Walz, M.; Stendel, M.; Bagrets, A.; Evers, F., Ab initio simulations of scanning-tunneling-microscope images with embedding techniques and application to C58-dimers on Au(111), *Phys. Chem. Chem. Phys.,* **2013,** 15, 6684-6690.
(19) Bagrets, A., Spin-polarized electron transport across metal-organic molecules: A density functional theory approach, *J Chem Theory Comput,* **2013,** 9, 2801-2815.
(20) Camarasa-Gómez, M.; Hernangómez-Pérez, D.; Evers, F., Spin–Orbit Torque in Single-Molecule Junctions from ab Initio, *J. Phys. Chem. Lett.,* **2024,** 15, 5747-5753.
(21) Camarasa-Gómez, M.; Hernangómez-Pérez, D.; Wilhelm, J.; Bagrets, A.; Evers, F., Molecular Transport, *arXiv preprint arXiv:2411.01680,* **2024**.
(22) Tkatchenko, A.; Scheffler, M., Accurate molecular van der Waals interactions from ground-state electron density and free-atom reference data, *Phys. Rev. Lett.,* **2009,** 102, 073005.
